# Expert consensus on Rezūm™: Indications, surgical technique and postoperative care

**DOI:** 10.1002/bco2.491

**Published:** 2025-02-15

**Authors:** Emilio López Alcina, Manuel Fernández Arjona, Ester Fernández Guzmán, Jorge Rioja Zuazu, Iván Schwartzman Jochamowitz

**Affiliations:** ^1^ Head of Service at the General University Hospital of Valencia Valencia Spain; ^2^ Head of the Urology Department at University Hospital of Henares Madrid Spain; ^3^ Assistant of the Urology Service of the Marqués de Valdecilla University Hospital Santander Spain; ^4^ Urology Specialist at Miguel Servet Hospital Zaragoza Spain; ^5^ Benign Prostatic Hyperplasia and Reconstructive Urology Unit at the Fundació Puigvert Barcelona Spain

**Keywords:** benign prostatic hyperplasia, lower urinary tract symptoms, minimally invasive surgical treatment, prostate, water vapour thermal therapy

## Abstract

**Background:**

Lower urinary tract symptoms associated to benign prostatic hyperplasia (LUTS/BPH) is a growing condition in males associated to a high clinical, economic and humanistic burden. Currently, there is a wide range of therapeutic options, both pharmacological and surgical. In recent years, several minimally invasive therapies have emerged, but they still have limitations. In this context, water vapour thermal therapy (WVTT), Rezüm™, is a new minimally invasive surgical technique with a low retreatment rate and the ability to preserve sexual function. The objective of this study is to establish recommendations for the correct execution of WVTT, as well as to define the ideal patient profile for the intervention.

**Methods:**

A literature search was conducted in PubMed, without date limitations, using the terms ‘benign prostatic hyperplasia’, ‘benign prostatic enlargement’, ‘prostatic hyperplasia’, ‘bph’, ‘Rezūm’, ‘water vapour thermal therapy’, ‘quality of life’ and ‘HRQOL’. A systematic review of the evidence was conducted and subsequently discussed in a face‐to‐face meeting with a panel of five experts in the field of urology. The aspects addressed were classified into patient profile, pre‐intervention, intervention and post‐intervention.

**Results:**

The search returned 172 results, ultimately leading to the analysis of 49 articles. Evidence and expert opinion showed that WVTT could be a good option for patients with moderate to severe symptomatology, no age restriction, Qmax <15 mL/s and prostate volume greater than 30 cm^3^, but not necessarily less than 80 cm^3^. Low retreatment rate and low impact on erectile capacity and ejaculatory function were also demonstrated.

**Conclusions:**

WVTT is considered a safe treatment option, even as a first‐line approach for certain patient profiles. However, further research is needed in areas where evidence and clinical experience remain limited, including postoperative catheter management, anaesthesia, antibiotic prophylaxis and follow‐up care for patients after the procedure.

## 1 INTRODUCTION

The term benign prostatic hyperplasia (BPH) is a histological diagnostic concept describing a hyperproliferative process of epithelial and stromal cells in the transitional zone of the prostate.^1^, ^2^ However, from a clinical point of view, BPH is a syndrome characterized by a benign enlargement of the prostate, which results in urinary outflow obstruction, and it is manifested with a range of symptoms known as lower urinary tract symptoms (LUTS).^2^ The histological prevalence of BPH is around 50% in men aged 60 years and can be as high as 90% over the age of 80 years,^2^, ^3^, ^4^, while LUTS prevalence varies from 44% in men aged 40–59 years to 70% in men over the age of 80 years. Moreover, the progressive ageing of the population could increase these figures even more in the coming years.^4^

The therapeutic approach focuses, rather than on BPH itself, on improving LUTS and the patient's quality of life (QoL), being the first step the modification of lifestyle habits and pharmacological treatment.^2^, ^3^ Surgical interventions are generally recommended in the case of failure to control LUTS or in the case of complications of the pathology in which surgery is necessary. However, there are many scenarios in which it can be considered from the early stages, such as the presence of comorbidities or the patients' own wishes, who sometimes refuse medication because they prefer to avoid prolonged use.^3^, ^5^ The gold standard technique is transurethral resection of the prostate (TURP), but this can lead to postoperative complications on ejaculatory function. As a result, so‐called minimally invasive surgical techniques (MIST) have emerged in recent years, which, although they have made advantages over TURP, their high retreatment rates, poor durability and some limitations in terms of treatment of the middle lobe mean that there are still certain restrictions on their use.^6^

In this context, Rezūm™ (water vapour thermal therapy, [WVTT]) is a MIST that, through radiofrequency, generates thermal energy, producing water vapour that destroys excess prostate tissue. As a result, obstructive symptoms are relieved without interfering with adjacent tissues, thus preserving sexual function.^3^, ^5^, ^7^ WVTT system is beginning to be adopted in urological practice, as reflected in the American Urological Association (AUA) and European Association of Urology (EAU) guidelines.^3^, ^5^ Numerous studies on the results of the WVTT exist to date, but there is no compilation of recommendations for its use, nor a discussion of it based on the clinical experience observed by experts in the field of urology, so in this paper, a systematic review of the existing evidence and discussion with a panel of experts will be conducted to develop a series of recommendations with the aim of establishing the appropriate patient profile who is a candidate for the technique, as well as the correct execution of the procedure.

## 2 MATERIALS AND METHODS

### 2.1 Expert selection and panel composition

The panel included five experts from different regions of Spain (Comunidad Valenciana, Aragón, Madrid, Cantabria and Cataluña), all of them in the field of urology, reconstructive urology and BPH. The experts had all collaborated with Boston Scientific and has vast experience in performing WVTT with Rezūm™ system, as well as in training new professionals in the technique, which was considered key for the discussion of the recommendations.

### 2.2 Literature search and recommendations formulation

Firstly, a literature search was conducted in PubMed, without limitations based on date or geographic zone, using the terms ‘benign prostatic hyperplasia’, ‘benign prostatic enlargement’, ‘prostatic hyperplasia’, ‘bph’, ‘Rezūm’, ‘water vapor thermal therapy’, ‘quality of life’ and ‘HRQOL’. Other data sources were also consulted, including relevant bibliographic citation lists from guideline development agencies (UCLA Integrated Staging System [UIIS], [AUA], [EAU] and National Institute for Health and Care Excellence [NICE]). The search yielded 172 results, from which only the meta‐analyses, systematic reviews and clinical trials were selected for the review (because of their high level of evidence), leaving the final number at 49.

Subsequently, a systematic literature review was undertaken, and a series of recommendations were made regarding patient profile, pre‐intervention, intervention and post‐intervention. A grade of recommendation and level of evidence based on the quality of the available evidence were assigned to each recommendation following the criteria listed in Table 1. When evidence was insufficient, only expert opinions were considered.

**TABLE 1:** Grades of evidence and levels of recommendation.

Grades of evidence	
1^++^	High‐quality meta‐analyses, systematic reviews of RCTs or RCTs with very low risk of bias
1^+^	Well‐conducted meta‐analyses, systematic reviews of RCTs or RCTs with low risk of bias
2^++^	High‐quality systematic reviews of case–control or cohort studies. High‐quality cohort or case–control studies with a very low risk of bias
2^+^	Case–control studies, cohort studies with low risk of bias
3	Non‐analytical studies, such as case reports and case series
4	Expert opinion
**Level of recommendation**	
A	At least one meta‐analysis, systematic review or RCT classified as 1^++^
B	A body of evidence including studies rated 2^++^
C	A body of evidence including studies rated 2^+^
D	Level 3 or 4 evidence

Abbreviation: RCT, randomized clinical trial.

### 2.3 Consensus meeting

The meeting took place on 11 December 2023, in Madrid to review the selected evidence and stablish the recommendations. Experts revised whether aspects extracted from the literature were consistent with what is observed in clinical practice, in order to develop a protocol for WVTT and establish the ideal patient profile for treatment with Rezūm™. In addition, the panel added several premises, not included in the previous analysis.

## 3 RESULTS

The conclusions obtained from the review of the 49 articles, and the subsequent discussions with the experts are summarized in Tables 2–4.

**TABLE 2:** Recommendations on the use of WVTT regarding patient profile.

Topic		Recommendations	Grade	Level evidence
**Patient profile**				
**Prostate volume**	**30–80 cm** ^**3**^	WVTT is a MIST that provides effective symptom relief and quality of life improvement that is maintained for more than 4 years in patients with a prostate volume between 30 and 80 cm^3^.^3^, ^5^, ^7^, ^8^	A	1^+^
	**>80 cm** ^**3**^	Micturition and symptomatologic parameters indicate that patients larger than 80 cm^3^ are also candidates for treatment.^9^, ^10^, ^11^, ^12^, ^13^	B	2^++^
**Prostate anatomy**		The results of WVTT are not influence by the area of the prostate being treated, making it suitable for application in both the lateral and middle lobes.^14^, ^15^	A	1^+^
**Age of the patient**		Age does not impact the benefits and risks associated with treatment, thus allowing its use without age‐related restrictions.^16^	B	2^++^
**Severity of symptoms**		WVTT is a favourable choice for men experiencing moderate to severe symptoms.^7^, ^8^, ^12^, ^15^	A	1^+^
**Micturion parameters**	**Qmax**	WVTT treatment can increase Qmax, maintaining improvement up to 4 years after treatment.^6^, ^7^, ^12^, ^14^, ^17^	B	2^++^
	**PVR**	In patients with BPH, a significant PVR (>150 mL) is not an exclusion criterion for the use of WVTT.	D	4
**Use in non‐responders' patients to pharmacological treatment**		WVTT may serve as a first‐line treatment option for patients who, upon being informed, opt out of pharmacological treatment, are non‐responders, experience side effects, are concerned about their sexual function and/or have multiple comorbidities.^18^, ^19^, ^20^, ^21^	B	2^++^
**Special considerations**		Pre‐intervention cystoscopy is recommended, especially in cases of suspected stenosis, bladder neck hypertrophy or endovesical lithiasis, among others.	D	4
**Pre‐intervention catheter‐dependent patients**		WVTT can successfully treat catheter‐dependent urinary retention, including middle lobe patients, after an initial failed TWOC and in an outpatient setting.^21^, ^22^, ^23^	B	2^++^
		Clinical evidence is scarce on this point. WVTT may be an option in selected patients with a catheter, but the benefit/risk balance should be discussed with the patient, taking into account the limitations of true MIST.	D	4
**Patient preferences and quality of life**		WVTT treatment may be considered in patients who prioritize their sexual well‐being and the reduction of side effects. The benefit/risk balance of this truly MIST should be adequately reported.^7^, ^14^, ^20^, ^23^, ^24^	A	1^+^

Abbreviations: BPH, benign prostatic hyperplasia; MIST, minimally invasive surgical techniques; PVR, postvoid residual volume; TWOC, test without catheter; WVTT, water vapour thermal therapy.

### 3.1 Patient profile considerations

#### 3.1.1 Prostate volume

The current indication in urological guidelines for WVTT is that the prostatic volume must be between 30 and 80 cm^3^.^3^, ^5^ The studies by McVary et al. and Roehrborn et al. were done under this premise, so there are positive results up to 4 years of follow‐up.^7^, ^8^ Several authors have included patients above this range, so the analysis of the available evidence for this population was also considered. Bole et al. compared the use of WVTT in patients with a mean prostate size of 119 cm^3^ versus mean sizes of 49 cm^3^ and concluded that subjects with prostate volumes >80 cm^3^ experienced symptomatic and objective improvements in the AUA Symptom Score (*p* = 0.04) and voiding parameters (*p* = 0.002) comparable to patients with glands smaller than 80 cm^3^.^9^ In a recent study, Woo et al. in a group of 47 patients reported prostate volumes ranging from 80.8 to 148.1 cm^3^. At 6 months, 83% of patients were treatment responders according to the primary efficacy end‐point, with an average International Prostate Symptoms Score (IPSS) improvement of 11.9 ± 7.5 points (95% CI lower bound of 9.7 points), indicating significant clinical benefit.^10^ In another current patient series, Elterman et al. demonstrated that patients with mean prostates volumes of 100.0 (88.5, 115.0) mL achieved a 59% improvement in IPSS at 12 months, indicating notable efficacy in symptom relief for large prostate glands.^11^ Moreover, in post‐marketing study, Darson et al., included patients up to 183 cm^3^ and separately analysed the subgroup with prostate >60 cm^3^, concluding that they had a similar probability as those in the <60 cm^3^ group of obtaining a clinically significant result of at least 3 points in the IPSS (94.7% vs. 78.9%, *p* = 0.11).^12^

All these studies were in line with the findings of the meta‐analysis conducted by McVary et al., which demonstrated that WVTT provides a statistically significant and clinically important short‐term improvement in LUTS with low complication rates in men with a prostate volume of at least 80 cm^3^.^13^

After discussion with the panel of experts, there was consensus on the appropriateness of the technique for sizes between 30 and 80 cm^3^ and for those >80 cm^3^. However, based on the clinical practice, it was noted that the experience obtained so far with large prostate volumes is insufficient, and, in these patients, the outcomes rely more heavily on the baseline urinary parameters rather than solely on the prostate volume.

#### 3.1.2 Prostate anatomy

WVTT can be used indifferently in either of the lobes, as well as in the middle lobe, in contrast to other therapies.^14^ Several studies include patients with middle lobe and the improvement in symptoms and flow appear to be independent of its presence,^15^ which was confirmed by the expert panel.

#### 3.1.3 Age of the patient

No age‐specific indication is available on the literature; however, Zhu et al. evaluated the results of WVTT in 146 patients <65 years versus 110 patients >65 years. Both groups showed significant improvements at 4 years in IPSS, QoL and Qmax (maximum flow rate) (*p* < 0.05), but no significant differences in either group at any follow‐up point. There was also no difference in the rates of either adverse events or retreatment (*p* = 0.86).^16^ The committee also noted no age‐related limitations in use.

#### 3.1.4 Severity of symptoms

The IPSS defines moderate symptoms as 8–19 points and severe symptoms as ≥19.^5^ In terms of clinical outcome, according to the AUA, patients with a decrease in IPSS of >3 points will be considered responders to treatment.^3^

In post‐marketing studies, Darson et al. evaluated 131 patients with moderate and severe symptoms treated with WVTT. Both groups, with similar prostate sizes, showed clinically and statistically significant improvements in IPSS at 3 and 12 months (*p* < 0.0001). A significant 6.1‐point reduction in IPSS (40.1%) was achieved in the moderate LUTS cohort at 3–6 months, which continued with a 40.6% reduction at 12 months from a mean baseline IPSS of 14.3 (*p* < 0.0001). In the severe LUTS cohort, there was a 14.4‐point reduction in IPSS (54.7%) from baseline at 3–6 months, which continued at 12 months with a reduction from 25.5 to 13.4 points (*p* < 0.0001).^15^ However, there were no significant differences in assessment time points between patients with moderate and severe symptoms.^8^ From this study, results were also obtained according to the presence of middle lobe and prostate size, showing a higher probability of having ≥3 points improvement in IPSS (91.7% vs. 75.4%, *p* = 0.03) in patients treated in the middle lobe, and 23 patients with prostates >60 cm^3^ were equally likely to have a significant clinical improvement of at least 3 points compared to patients <60 cm^3^ (94.7% vs. 78.9%, *p* = 0.11).^12^ In the same way, but with higher temporalities, McVary et al. concluded that men with moderate and severe symptoms experience symptomatic relief, with similar improvements in IPSS at 4 years, 46.1% and 46.9%, respectively.^7^

#### 3.1.5 Micturition parameters

To date, the two parameters most frequently evaluated in clinical trials are maximum flow rate (Qmax) and postvoid residual volume (PVR). Regarding Qmax, no specific value has been identified to establish it as an inclusion criterion, although the tendency is to select patients with Qmax <15 mL/s. In the study by Darson et al., after 12 months, there was a significant improvement in Qmax values (*p* < 0.0001) in both moderate and severe symptom groups.^12^ Similarly, the original 4‐year clinical trial by McVary et al. showed an increase in Qmax of 45% in the moderate symptomatology group and 51.3% for severe symptomatology (*p* < 0.0001).^7^ However, while Garden et al. found significant improvements in the >80 cm^3^ prostate group (from 7.39 to 14.60 mL/s; *p* = 0.039), this was not the case in the <80 cm^3^ group (9.47–10.90, *p* = 0.187).^14^ In meta‐analyses, Qmax showed significant improvement at all follow‐up points, with no differences mentioned for any other parameter.^6^, ^17^

Regarding PVR, studies with a duration of 3 months demonstrated a notable reduction of 74% (from 197 ± 150 to 51 ± 22 mL)^35^ and in those up to 4 years, a decrease of up to 85.7%.^25^ In those analysing PVR by prostate size, although more profound changes were seen in the >80 cm^3^ group (161.09–80.85 mL, *p* = 0.009) than in the <80 cm^3^ group (89.51–62.72, *p* = 0.027), both improvements were statistically significant.^14^

Experts agreed that an improvement in both parameters is observed after treatment with WVTT, although it was considered that there is not sufficient evidence to limit the application of the technique depending exclusively on the patient's PVR nor to define a PVR threshold that would restrict its use.

#### 3.1.6 Use in non‐responders' patients to pharmacological treatment

Pharmacological treatment is the first choice for treating BPH. Nonetheless, its efficacy is limited and adverse effects cause discontinuation in more than 15% of cases.^18^, ^19^ For that reason, patient selection in studies with WVTT usually involves patients whose medical treatments have been ineffective, poorly tolerated or refused.^20^, ^21^

While the panel acknowledged the recommendation, they deemed it essential to incorporate certain nuances. Several recommendations suggest WVTT as an alternative to medication, particularly in younger individuals, a scenario that is not consistently reflected in actual practice.

#### 3.1.7 Pre‐intervention catheter‐dependent patients

Regarding catheter‐dependent patients, Elterman et al. conducted an analysis of 17 catheter‐dependent patients with a mean prostate volume of 84.4 cm^3^, with or without the presence of the middle lobe. All patients had at least one recent failed test without catheter (TWOC). At 1 month, 13/14 patients had voided spontaneously and were not catheterized; at 3 months, 14/14 and at 6 months, 15/15 (one patient was lost to follow‐up).^22^ Another study with 49 patients, all catheter‐dependent, with a mean volume of 73 cm^3^ and 80% mid‐lobe presence, showed a success rate in terms of catheter‐dependent urinary retention of 87.8% at 6 months. Moreover, a significant improvement in IPSS of 13 points on average was observed at Month 6 (17 points pretreatment to 4 points; *p* < 0.01).^23^ In the Winkler et al. trial, 26 of 92 patients were catheter‐dependent pre‐intervention, and all of them were catheter‐free at 6 weeks.^21^

In the discussion, the experts noted that in these patients, the results of the clinical trials can be misleading, as the aim is not only to remove the catheter but also to test urinary function after a period of time following catheter removal. In clinical practice, this patient profile exhibits poorer urination patterns compared non‐catheter‐dependent patients, resulting in outcomes that may not be as favourable. It is essential to communicate this information to patients in advance.

#### 3.1.8 Patient preferences and QoL

The indicator related to QoL in multiple studies is the IPSS‐QoL. Evidence suggests that WVTT can significantly improve QoL in men with moderate to severe LUTS by 43% (*p* < 0.0001) from Month 3 and sustained over 4 years. Patients who underwent middle lobe treatment showed similar objective and subjective improvements to those who did not,^7^ and those who were catheter‐dependent pre‐intervention also showed a statistically significant decrease of 4 points on average (*p* < 0.01) at 6 months.^23^

In terms of patient preferences when choosing treatment with WVTT, there is little evidence collected. Some studies mention that the main reason given is to maintain sexual function.^20^, ^24^ In Garden et al., patients with prostate volume >80 cm^3^ were informed that the indication for the technique was only for sizes <80 cm^3^, yet they chose WVTT as a less invasive treatment, as an alternative to continuing other medical treatments, and mitigated sex‐related side effects.^14^

Based on expert's experience, the greatest concern that patients report is the fear of complications and, to a lesser extent, the subsequent catheterisation time, and hospital stay.

#### 3.1.9 Special considerations

Experts thought it appropriate to make an additional contribution, concerning the performance of cystoscopy. All considered that it can be helpful to have more detailed information on prostate size and morphology, particularly when a special condition is suspected.

Although not all trials mention cystoscopy prior to the use of WVTT, several of them discuss the usefulness of this practice.^7^, ^35^

The patient profile recommendations are summarized in Table 2.

### 3.2 Procedural considerations

#### 3.2.1 Pre‐intervention medication of the patient

##### Anticoagulants and antiplatelet agents

In the current literature, limited guidance exists on medication management for patients undergoing WVTT. Nonetheless, there are specific studies regarding anticoagulants and antiplatelet agents. Bausch et al. trial looked at the subgroup of subjects undergoing these treatments. Eighty‐three out of 211 patients were on medication and underwent surgery without a pause in treatment, either with anticoagulants or platelet aggregation inhibitors. Of these, 45 were on acetylsalicylic acid, two on clopidogrel, five on acetylsalicylic acid and clopidogrel, one on heparin, eight on coumarins and 22 on direct factor Xa inhibitors. No bleeding complications occurred, so it was assumed that WVTT is a safe technique.^25^ It was further noticed that management in clinical practice is quite variable. In some cases, acenocoumarol is replaced by heparins; in others, anticoagulation is interrupted in the days prior to the intervention. This variability stems from the limited literature and clinical experience available; however, there was consensus that the technique would allow this type of treatment not to be discontinued.

##### Other medication

Patients sometimes undergo preoperative washouts of treatments such as antihistamines, antispasmodics, alpha‐blockers, anticholinergics, 5‐alpha reductase inhibitors, phosphodiesterase‐5 inhibitors or androgen suppressants.^26^, ^27^, ^28^ Not performing these washes could lead to smaller improvements in absolute IPPS values, as suggested by Mollergarden et al.^15^ On the other hand, data are available from a study of 129 patients 85 of 95 who were on alpha‐blockers and/or 5‐alpha reductase stopped taking them 180 days after the intervention.^29^ Nonetheless, other studies found a significant decrease in the use of alpha‐blockers but no significant change in the use of either 5‐alpha reductase inhibitors or phosphodiesterase‐5 inhibitors.^14^ Finally, available data in the pre‐intervention group of catheter‐dependent patients indicate that, in a study of 38 patients, 26 were on some form of medication, and 18 of them stopped at the end of the study.^30^

##### Antibiotic prophylaxis

According to local guideline recommendations, antibiotic prophylaxis was administered to all patients. One of the studies included quinolones (levofloxacin 500 mg/day for 7 days) or cephalosporin 400 mg/day for 7 days,^31^ while another used ciprofloxacin from the night before until 5 days after.^30^ It was pointed out that despite the lack of evidence, a prior culture should be conducted to detect cases of asymptomatic bacteriuria, for which antibiotic prophylaxis is always recommended.

#### 3.2.2 Intra‐intervention: Surgical technique

##### Anaesthesia

There are no specific recommendations regarding WVTT intervention. Guidelines state that it is an outpatient surgical procedure that can be performed under general anaesthesia or local anaesthesia with sedation.^3^ In a post‐marketing study of 131 patients, 86% received intravenous sedation, 15% general anaesthesia and 6% prostate block. All procedures were performed in the office or outpatient setting.^12^ Other studies report that the type of anaesthesia depends on local protocols and patient preferences.^31^

Of note are the results of a study that evaluated the efficacy of local anaesthetic prostate blocks during WVTT intervention, comparing one group who underwent the indicated technique versus another who did not, both in the context of intravenous sedation. No significant differences were found neither in markers of postoperative pain between the groups, leading to the conclusion that when conscious sedation is performed, a local anaesthetic prostate block is not necessary.^32^

Finally, evidence from patient series has been reported on the use of transurethral intraprostatic anaesthesia (TUIA) during WVTT treatment, highlighting favourable outcomes. This technique has been shown to provide effective pain management, with median pain scores reported between 0 and 3 on a numeric rating scale, minimal postoperative discomfort and an absence of perioperative complications. Furthermore, it demonstrated a reduction in operative and hospitalization times, underscoring its safety and efficiency, particularly in patients for whom general anaesthesia is not suitable.^33^

Experts highlighted that the availability of resources is crucial when choosing the type of anaesthesia; however, sedation is the preferred technique.

##### Number of injections

The number of injections in each prostate lobe is determined by the length of the prostatic urethra, adapting to the morphology of the hypertrophied gland and may include the middle lobe or enlarged central area.^7^ In the clinical trial by McVary et al., the mean number of injections performed was 4.7 ± 1.7 and 1.6 ± 0.7 in those patients with mid‐lobe.^7^ In clinical trials where only patients with prostates smaller than 80 mL were recruited, an average of 4.7 ± 1.7 injections (with an average prostate size of 45.8 ± 13 mL)^7^ and 4.9 injections (with an average size range of 58–44.82 mL) were recorded, with a maximum of 10 treatments.^34^ In studies where only prostate sizes over 80 mL were included, the average number of injections was 10.6, with an average prostate size of 92.9 mL (range: 85.1–115.6 mL).^10^ In both groups, patients with a middle lobe presented an average of 1.6 injections, with a maximum of two injections. In line with these findings, Garden et al. conducted a study that included patients with prostate sizes both below and above 80 mL. On average, subjects with prostate >80 cm^3^ received 9.61 injections compared to 4.76 in the <80 cm^3^ group. This trend was also seen when the middle lobe was present.^14^ Some authors have considered the concept of the number of injections in relation to prostate volume. The work of Ines et al. included patients with prostates from 20 to 120 cm^3^. Despite the total number of injections increasing proportionally with size, there are a consistent number of treatments administered to each side lobe for every 10 cm^3^ of volume.^29^

On the other hand, one study reports cases where the number of injections exceeded the 15 indicated. Four patients in the group of subjects with a prostate >80 cm^3^ received 16, 16, 17 and 18 injections, but without mentioning anything in terms of safety or efficacy.^14^ In addition to the number of injections, experts consider important to take into account the anatomy of the prostate.

##### Distances

The tip of the needle should be visually positioned and initially inserted 1 cm from the bladder neck, into transitional and central prostatic adenomas. Intravesical prostatic bulges and the middle lobe must be injected starting 1 cm from the edge of the bulge. The needle must be retracted after each steam injection and repeated in 1 cm increments from the anterior point to the prostatic tissue just proximal to the *verumontanum*.^7^

##### Learning curve

The addition of this concept was prompted by expert input, as there is currently no published information available on this topic. The learning curve for Rezūm™ is fast; however, it should be noted that the procedure is more complex than it initially appears. For this reason, it is recommended to perform the first interventions with a trained professional, to avoid that an incorrect use in the initial phase of the curve creates the impression that the technique is not effective. It is also important to perform the punctures correctly. It is recommended to start with small prostates, as these only have a single possible injection line. Large prostates either have several or even have to be injected in a *zigzag* pattern. Finally, it is necessary to communicate that the use of WVTT is not a short‐term option but should be considered as a long‐term alternative and thus be introduced into the service portfolio. To this end, it would be useful to develop a training programme.

The recommendations on the technique's execution are summarized in Table 3.

**TABLE 3:** Recommendations on the use of water vapour thermal therapy (WVTT) regarding procedure.

Topic		Recommendations	Grade	Level evidence
**Procedure**				
**Medication**	**Anticoagulants and antiplatelet agents**	It may not be necessary to pause treatment with anticoagulants or antiplatelet agents for an intervention with WVTT.^25^	C	2^+^
		Despite the limited published evidence, the potential benefits of this technique in patients on anticoagulants and antiplatelet therapy are substantial.	D	4
	**Other medication**	Discontinuation of any drug treatment would not be necessary for reasons directly related to the technique.^14^, ^15^, ^26^, ^27^, ^28^, ^29^, ^30^	C	2^+^
**Antibiotic prophylaxis**		No single criterion for antibiotic prophylaxis is available and can be adapted to the judgement of the specialist.^30^, ^31^	C	2^+^
		Pre‐culture and targeted prophylaxis are recommended in patients with asymptomatic bacteriuria.	D	4
**Anaesthesia**		There is no single criterion for anaesthetic technique, and it can be adapted to both specialist judgement and patient preference.^3^, ^12^, ^31^, ^32^, ^33^	C	2^+^
		WVTT is a technique that can be safely performed outside a conventional operating theatre.	D	4
**Learning curve**		Although accreditation is required to perform this procedure, it is advisable to undertake the first few treatments under the guidance of an expert and to apply it in cases of low complexity, in order to consolidate the technical details of the procedure. In addition, it is recommended to visit reference centres where the technique is already implemented.	D	4
**Number of injections**		The number of injections increases proportionally to the size of the prostate, considering the anatomy of the prostate.^7^, ^10^, ^14^, ^29^, ^34^	B	2^++^
**Distances**		The technique should be performed as indicated in the instructions.^7^	A	1^+^

### 3.3 Post‐intervention considerations

#### 3.3.1 Retreatment rate

Regarding retreatment, studies reported 2‐ and 4‐year retreatment rates of 3.7% and 4.4%, respectively.^7^, ^8^ In the 2‐year data, eight patients had to be retreated, three of them with WVTT. However, six of the eight cases were related to middle lobes that had not been treated initially.^8^ In the 4‐year study, six out of 135 subjects (4.4%) had to be retreated, due to lack of treatment of the middle lobe in 4/6 cases, thus, decreasing the retreatment rate to 2.2%.^7^

Relating to prostate size, a study with patients with prostates <80 cm^3^, the rate was 4.76%, similar to the other trials, while in the >80 cm^3^ group, it rose to 8.33%. In this study, the authors noted that there was a possibility that these rates were under‐reported, as some planned activities were cancelled during the pandemic.^14^ Finally, the meta‐analysis by Miller et al. confirms the idea that WVTT treatment shows low retreatment rates at 4 years.^17^

#### 3.3.2 Type and duration of the urethral catheter

WVTT guidelines recommend leaving a urinary catheter in place for 5–7 days after surgery.^3^ Some investigators, such as Haroon et al., followed the indication;^35^ however, in Winkler et al., the mean length of stay of patients in hospital after the intervention was 3.43 ± 1.73 days. A total of 66 (71%) of patients were already discharged with the catheter removed, 18.5% left the hospital with a suprapubic catheter and 9.8% with a transurethral catheter. All catheter types were removed in an outpatient setting at 6 weeks.^21^ There are other studies where investigators preventively prefer longer times to avoid cases of acute urinary retention (AUR), with a median of 7 days after surgery. In the case of this study, however, there were 16 cases (11.8%) of AUR, of which 13 resolved with an indwelling catheter for 3 days (median).^31^

Experts highlighted the potential role of suprapubic catheterization as an alternative to prolonged postoperative catheterization, particularly in patients with hypotonic or neurogenic bladders. Additionally, they noted the potential utility of intermittent self‐catheterization for patients unable to void after the procedure.

According their observations in clinical practice, catheterisation time can be longer than 5–7 days, varying according to prostate size, and, therefore, the number of injections. Furthermore, in patients with a pre‐intervention catheter, they recommend extending this period even further.

#### 3.3.3 Temporary prostatic stent (TPS)

TPS, such as the Spanner® type, has also been used in some cases. Two studies report results. Mooney et al. found that stented patients had a lower rate of urinary retention (13% vs. 16.3%) than catheterised patients but increased urinary tract infections (43.5% vs. 13.4%).^30^

In another study, stents were used for all non‐anticoagulated patients. Men with a catheter carried a mean of 4.4 ± 4.3 days, while the mean with the stent was 19.3 ± 12.6 days. Of the 45 stented patients, five were unable to micturate immediately after stent placement and were replaced by a catheter. In the analysis of results, they reflect that there was no significant difference in IPSS in the 91–180‐day interval between the stent and catheter groups, although there were more cases of infections in the stented patients (23.7% vs. 14.6%), with no significant difference (*p* = 0.31). On the other hand, the TPS group showed a retention rate of 2.06% versus 14.0% for the catheter group (*p* = 0.11).^15^

Based on their experience in routine clinical practice, the expert panel indicated that it has been used in some cases and that its use is quite comfortable, seeing it as an alternative to bladder catheterisation that can be agreed with patients.

#### 3.3.4 Postoperative recommendations

Although not reported in the literature, the experts discussed several considerations to take into account after the technique is performed in order to minimize subsequent complications as much as possible.

#### 3.3.5 Patient follow‐up

Experts considered that, as the expected reduction in prostate size is achieved after 3 months, a comprehensive assessment with ultrasound, flowmetry, PSA and IPSS should be performed between 3 and 6 months. In addition, the introduction of validated quality of life questionnaires is essential, as this is the main objective of the treatment.

### 3.4 Adverse events

In the original randomized study by McVary et al. the adverse events reported were dysuria (16.9%), haematuria (11.8%), pollakiuria and urinary urgency (5.9%), AUR (3.7%) and suspected urinary tract infection (3.7%); all were routinely treated or resolved without treatment within 3 weeks.^7^

Two subsequent meta‐analyses reported the same adverse events. Yang et al. obtained the following results: dysuria (21%), haematuria (14%), urinary retention (12%), haematospermia (10%), pollakiuria (10%) and urinary urgency (10%); most of them occurred within 3 months after surgery and resolved within 3 weeks.^6^ Miller et al. reported only those with more than 5%: dysuria (16.2%), urinary retention (11.2%) and urinary tract infection (10.9%).^17^

In terms of drawbacks related to patients' sexuality, a randomized controlled study was conducted in 2016 to study erectile and ejaculatory function in patients undergoing WVTT, comparing at 3 months with the blinded arm and following the active arm up to 12 months. No de novo treatment‐ or device‐related erectile dysfunction occurred after heat therapy. The International Index of Erectile Function (IIEF) and Male Sexual Health Questionnaire for Ejaculatory Function (MSHQEjD) scores were not different from those of the control group at 3 months or from baseline at the end of the year. The ejaculatory discomfort score improved by 31% from baseline (*p* = 0.001). In addition, 32% of subjects achieved minimal clinically important differences in erectile function scores at 3 months, and 27% at 1 year, including those with moderate to severe erectile dysfunction.^28^ Although later investigations obtained similar results, Mollergarden et al., reported 4/129 patients with erectile dysfunction and 4/129 with anejaculation, although they indicate that the absence of assessment of erectile function at baseline means that the relationship is not entirely certain.^15^

The meta‐analysis by Yang et al. indicated that changes in IIEF and MSHQEjD were not significant in patients treated with WVTT. Ejaculatory discomfort decreased significantly by 0.75 points after 1 year, and, in contrast to TURP, no de novo erectile dysfunction or ejaculatory dysfunction was detected.^6^

Finally, studies have also been conducted in patients stratified into two groups: those meeting randomized controlled trial inclusion criteria and those with broader clinical profiles, including individuals with larger prostate sizes, indwelling catheters, antiplatelet or anticoagulant therapy and higher comorbidity burdens. It was demonstrated that WVTT provided a safe and effective treatment option, with significant improvements in urinary and sexual outcomes observed across both groups. These results underscore the robustness of the therapy and its applicability to diverse patient populations with BPH^36^

Postoperative recommendations are summarized in Table 4.

**TABLE 4:** Recommendations on the use of WVTT regarding post‐intervention.

Topic	Recommendations	Grade	Level evidence
**Post‐intervention**			
**Retreatment rate**	With the evidence available so far, treatment with WVTT has a low retreatment rate, as long as the middle lobe is considered for early intervention.^7^, ^8^, ^14^, ^17^	A	1^+^
**Type and duration of the urethral catheter**	Post‐intervention catheter duration ranges from 3 to 10 days.^3^, ^21^, ^31^, ^35^	B	2^++^
	Postoperative catheterization time appears to be longer than initially observed in clinical trials. It is recommended to adapt the catheterisation time according to the number of injections and prostate size. It is recommended to extend the duration in patients with pre‐intervention catheterization.	D	4
**Temporary prostatic stent**	Due to the few conclusions to be drawn from the literature and because of the limitations observed, the use of this device is left open to the discretion of the clinician. It can be used as an alternative to conventional bladder catheterisation.	D	4
**Adverse events**	Most of the adverse effects that arise with WVTT are transient and CLAVIEN‐DINDO <3. In addition, the non‐significant impact on erectile capacity and ejaculatory function makes it an attractive option for sexually active patients.^6^, ^7^, ^15^, ^17^, ^28^, ^36^	A	1^+^
**Post‐operative recommendations**	The use of corticosteroids, such as deflezacort or intramuscular betamethasone, or anti‐inflammatory drugs (NSAIDs) is recommended 10–15 days after surgery.	D	4
	As with other MIST, it is advisable to monitor urinary pH to assess whether urine alkalinisation with bicarbonate or potassium citrate is necessary to prevent retention and control dysuria; maintain the patient's pre‐intervention medication for BPH for 1 month; and finally, prescribe prophylactic antibiotic treatment during the catheterisation period.	D	4
**Patient follow‐up**	As a nonstandard procedure, patient follow‐up should be close in relation to other established treatments.	D	4
	As the expected reduction in prostate size is achieved after 3 months, a comprehensive assessment with ultrasound, flowmetry, PSA and IPSS should be performed between 3 and 6 months. In addition, the introduction of validated quality of life questionnaires is essential, as this is the main objective of the treatment.	D	4
	As with other techniques, adjust medication for filling symptoms according to the patient's progress.	D	4

Abbreviations: BPH, benign prostatic hyperplasia; IPSS, International Prostate Symptoms Score; MIST, minimally invasive surgical techniques; PSA, prostate specific antigen; WVTT, water vapour thermal therapy.

## 4 CONCLUSIONS

The systematic literature review conducted, with subsequent expert panel consensus, positions the WVTT, Rezūm™, as a new MIST for patients with moderate to severe symptomatology, no age restriction, Qmax <15 mL/s and prostate volume greater than 30 cm^3^, but not necessarily less than 80 cm^3^. Previous catheter carriers will also be candidates, although they tend to have somewhat worse outcomes because their baseline condition is more compromised. Moreover, its low retreatment rate and its low impact on erectile capacity and ejaculatory function make it a safe option, even as a first line of treatment, for patients who are concerned about their sex life or who, having been previously informed, refuse pharmacological treatments. However, it remains imperative to conduct additional research and studies in less explored areas where current evidence and expertise are lacking, including postoperative catheter management, anaesthesia, antibiotic prophylaxis and follow‐up care for patients after the procedure.

## AUTHOR CONTRIBUTIONS

All the authors actively participated in the consensus process and equally contributed to the manuscript revision and approval of the content.

## CONFLICT OF INTEREST STATEMENT

Authors have received honoraria for their assessment and consultation services from Boston Scientific Ibérica, S.A.
